# The practices and beliefs of dental professionals regarding the management of patients taking anticoagulant and antiplatelet drugs

**DOI:** 10.1038/s41405-022-00127-3

**Published:** 2023-01-25

**Authors:** Niamh Kelly, Laura Beaton, Jennifer Knights, Douglas Stirling, Michele West, Linda Young

**Affiliations:** 1 Dental Core Trainee, Dundee Dental Hospital and School, NHS Tayside, Dundee, UK; 2 NHS Education for Scotland, Scotland, UK

**Keywords:** Warfarin in dentistry, Oral surgery

## Abstract

**Aim:**

This study aimed to inform the implementation of the updated Scottish Dental Clinical Effectiveness Programme (SDCEP) guidance, ‘Management of Dental Patients taking Anticoagulant or Antiplatelet Drugs’, and to determine training needs by investigating dental professionals’ current practice and beliefs regarding management of patients taking these medications.

**Methods:**

Dental professionals were recruited via the NHS Education for Scotland Portal. The online questionnaire collected demographic information, data on current practice and information about beliefs regarding behaviours related to the management of patients on anticoagulant or antiplatelet medication. Quantitative data were analysed using SPSS and subjected to frequency calculations, t-tests, one-way ANOVA and linear regression. Qualitative data were collected via free text boxes and analysed using thematic analysis.

**Results:**

One hundred and fifty-seven participants responded to the questionnaire. The majority of respondents stated they were aware of the guidance and always based their practice on it. The majority of respondents always assessed the patient’s individual bleeding risk prior to dental procedures. Most respondents felt that they did not know how to appropriately manage patients taking low doses of low molecular weight heparins (LMWH), and only 38% of respondents always followed SDCEP guidance about direct oral anticoagulants (DOAC) medication and procedures with a low associated risk of bleeding.

**Discussion:**

This study demonstrates a need for further educational support surrounding LMWHs and management of patients on DOAC medication. Time and remuneration represent barriers to guidance implementation in primary care.

**Conclusion:**

There is good awareness and adherence to the guidance in primary care settings, however training needs were identified to support implementation.

## Introduction

Medical conditions such as atherosclerosis or cardiac arrhythmias increase patients’ risk of thrombosis, resulting in potentially fatal events such as cardiac arrest, stroke or pulmonary embolism [1]. Antiplatelet and anticoagulant medication are prescribed to reduce this risk for patients with mechanical valve replacement, cardiac stents, atrial fibrillation, a history of stroke or cardiovascular incidents or arrhythmias [2–4]. However, the use of anticoagulant and antiplatelet medication increases bleeding risk; therefore, an assessment of bleeding risk prior to surgical procedures, such as those carried out in dentistry, is fundamental.

The Scottish Dental Clinical Effectiveness Programme, (SDCEP) produced guidance in 2015 to provide support surrounding the management of dental treatment for patients on antiplatelet and anticoagulant medication. Since the publication of the 2015 guidance, the landscape of anticoagulant and antiplatelet use has changed.

Anticoagulant medication is prescribed for approximately 1.25 million people per year in the UK [5], with the use of direct oral anticoagulant (DOAC) medication increasing from 16% in 2015 to 62% of all anticoagulant medication by 2019 [6]. Warfarin has been used for over 60 years, however in the last 8 years its use has decreased, due to the availability of DOACs [7]. Although the use of antiplatelet medication has remained relatively stable [8], the introduction of newer drugs, such as prasugrel and ticagrelor, has resulted in a greater variation in antiplatelet drugs that patients may be prescribed [9]. Although less commonly used than oral anticoagulants or antiplatelet agents, parenteral anticoagulants such as the low molecular weight heparins (LMWHs) may also be encountered.

Prescribing of anticoagulant and antiplatelet medication may continue to increase, as the prevalence of patients with cardiovascular and chronic cerebrovascular illnesses continues to rise, given an aging population. Therefore, dental professionals are likely to encounter these patients more frequently [10, 11].

Although anticoagulants such as warfarin and antiplatelet drugs such as aspirin and clopidogrel have been widely used for a number of years, with various dental guidelines relating to their use [2, 12], less evidence-based guidance has been available for DOACs such as apixaban, rivaroxaban, dabigatran and antiplatelets such as prasugrel and ticagrelor [9].

The 2015 SDCEP guidance ‘Management of Dental Patients Taking Anticoagulants or Antiplatelet Drugs’ provides recommendations and clinical advice for managing dental patients taking anticoagulants or antiplatelet medication including the newer drugs. Despite this, variation in clinical practice between general dental practitioners, lack of confidence and failure to follow an evidence-based approach in the management of these patients has been reported [13].

SDCEP developed an update of its guidance, which was published in 2022, taking the changed drug prevalence and other developments into account. The guidance update includes the newest DOAC edoxaban, new recommendations for managing patients taking LMWH, and updated information to support bleeding risk assessment.

The aim of this paper is to detail work undertaken by TRiaDS (Translation Research in a Dental Setting) to support the implementation of the updated SDCEP guidance through determining barriers to compliance and identifying professional training needs. Dental professionals were recruited from a range of clinical settings such as primary dental care, community dental services and hospital dentistry in Scotland, to highlight barriers to implementation in primary practice, to determine how this may be addressed.

## Methods

### Sample and design

An online cross-sectional survey was used to gather data. NHS and private dental practitioners, dental therapists, and dental hygienists in both the General Dental Service (GDS) and Public Dental Service (PDS) in Scotland were recruited via the NES Portal, an online tool used for course bookings/management administered by NHS Education for Scotland. Only those who had previously opted in to receive marketing correspondence were included in the dissemination of the questionnaire. Vocational Dental Practitioners in Scotland were recruited through the Dental Vocational Training Hub.

### Data collection

The questionnaire was hosted in Questback, an online survey tool, and disseminated in December 2021. The survey was open to responses until January 2022. Reminders to complete the survey were provided 2 weeks before the closing date.

### Questionnaire development

The online questionnaire collected demographic information, data on current practice and information about beliefs regarding taking a medical history, assessing bleeding risks, managing patients taking DOAC medication, managing patients taking warfarin or another vitamin K antagonist (VKA), managing patients taking a LMWH, carrying out haemostatic packing and suturing and referrals to secondary care for patients taking anticoagulant or antiplatelet drugs. Free text boxes were also provided, to allow respondents to explain their answers in more detail.

The COM-B model [14] was used as a theoretical base to inform questionnaire development. COM-B is a model used to understand behaviour in the context in which it occurs [14]. It investigates capability, opportunity, motivation, and behaviour, positing that for a behaviour to occur, the person(s) involved in the behaviour must have the capability, opportunity and motivation to do it. The categories assessed are further subdivided into physical capability, psychological capability, physical opportunity, social opportunity, reflective motivation, and automatic motivation. Questions were developed for each behaviour, addressing each of these subcategories.

### Data analysis

Items comprising each COM-B domain were scored positively, summed when appropriate and an average ‘domain score’ calculated for each respondent. Cronbach’s alpha and Spearman Brown tests were used to test reliability for each domain and behaviour. An alpha score of 0.6 was considered satisfactory for reliability [15]. Descriptive frequencies were recorded for the quantitative data, including current practice, beliefs and demographics.

Statistical analysis was carried out using t-tests, one-way ANOVA and Tukey post-hoc tests to explore the relationship between the behaviours and demographic information. Statistical significance was set at *p* < 0.001, to account for multiple testing [16]. Linear regression was used to explore the relationships between the behaviours and the COM-B domains. In addition, demographic information (i.e., sex, professional role, age, location, setting and years since qualified) was included in the regression model to control for confounding factors.

Qualitative analysis of free-text responses was conducted using thematic analysis. Thematic analysis is a method of qualitative data analysis, involving a six-step process: analysis of the data, generating initial codes, searching, reviewing and defining themes, and producing a summary report [17, 18]. Thematic analysis was carried out by 3 authors independently. Analysis was completed by NK, with support from LB and JK; any disagreements regarding coding were discussed until consensus was reached. A six-point framework [17] was used to ensure validity and reproducibility of the process.

#### Ethical considerations

Given that this project constituted part of the process of SDCEP guidance production and implementation, ethical approval was not deemed necessary. Completion of the questionnaire represented consent to take part. A participant information sheet was provided in the recruitment email outlining the purpose of the survey and advising that participation was voluntary.

## Results

One hundred and fifty-seven questionnaire responses were submitted. Seventy-five percent of respondents were female and 25% were male. Fifty-one percent of participants were between 30 and 50 years old.

Seventy-four percent of respondents worked in the GDS, 22% worked in the PDS, and a small number of participants, 4%, worked in other settings such as military dental services, private practice and hospital dental services. The majority of respondents (81%) worked in cities and towns, whilst 19% worked in remote and rural settings.

Job roles included: principal dentists (15%), associate dentists (53%), vocational dental practitioners (13%), and hygienists (1%). Eighteen percent of participants worked in ‘other’ job roles as consultants, specialists, specialty dentists, civilian dental officers, and clinical director roles (Table 1).

**Table 1:** Respondent demographics.

Demographic		*n*	Percentage (%)
Sex	Male	38	25%
	Female	115	75%
	Prefer not to say	1	1%
Location	Rural	30	19%
	Towns/Cities	127	81%
Age	20–29	32	21%
	30–39	37	25%
	40–49	40	27%
	50–59	36	24%
	60–69	6	4%
Setting	GDS	116	74%
	PDS	34	22%
	Other	6	4%
Job role	Principal	24	15%
	Associate	82	53%
	VDP	20	13%
	Hygienist	2	1%
	Other	28	18%

*GDS* general dental services, *PDS* public dental services, *VDP* vocational dental practitioner.

*Due to rounding of results, percentages may not equal 100%.

### Prevalence

Fifty-four percent (*n* = 84) reported that they usually saw patients taking antiplatelets weekly. Thirty-three percent (*n* = 52) reported they saw patients taking DOAC medication weekly and 11% (17) saw patients taking vitamin K antagonists (VKA) weekly. Although 83% (*n* = 130) of dental professionals always asked for a list of medications when completing a medical history form, only 59% (*n* = 93) reported that they always asked specifically about the use of anticoagulant or antiplatelet medications.

LMWHs were less commonly encountered by respondents, with 23% (*n* = 35) stating they never encountered patients on a LMWH and 5% (*n* = 8) of respondents encountering patients on a LMWH at least once a week. A further 9% (*n* = 14) were unaware if patients they treated were taking a LMWH.

### Current Practice

All participants who responded to this question (*n* = 156) were aware of the 2015 SDCEP guidance on the management of dental patients taking anticoagulant or antiplatelet medication, and 77% (*n* = 121) of respondents answered that they always based their practice upon the SDCEP guidance.

For patients on antiplatelet or anticoagulant medication, 78% (*n* = 123) of respondents always assessed individual bleeding risk prior to dental procedures, and 90% (*n* = 142) always assessed the risk of bleeding complications associated with the required dental procedure.

Only 38% (*n* = 56) of respondents stated they always followed SDCEP guidance to treat patients taking a DOAC without interrupting their medication regime for procedures with a low risk of bleeding complications. For procedures with a higher risk of bleeding associated, 48% (*n* = 73) of respondents always asked patients on apixaban or dabigatran to miss their morning dose and 41% (*n* = 61) of respondents always advised patients on rivaroxaban or edoxaban to delay their morning dose.

For patients on warfarin or another VKA, 97% (*n* = 151) of respondents ensured the INR (International Normalised Ratio) had been checked within 72 hours of the procedure if stable, in accordance with SDCEP guidance and 82% (*n* = 129) checked no more than 24 hours prior to the procedure if unstable, in accordance with SDCEP guidance.

For patients on a LMWH requiring a dental procedure with a low associated risk of bleeding complications, 22% (*n* = 32) of respondents always treated without interrupting their anticoagulant medication. Thirty-one percent (*n* = 49) of respondents always carried out packing and suturing for procedures likely to cause bleeding for patients taking anticoagulant or antiplatelet medication. Sixty-one percent (*n* = 96) of respondents stated that if they had concerns about safely treating a patient in primary care, they would first contact a colleague in secondary care to discuss the most appropriate management, before making a referral.

There were no statistically significant differences noted between respondents’ current practice behaviours and their demographic profile.

## Beliefs of respondents

### Taking a medical History

All (*n* = 157) respondents agreed that they understood the reasons for asking about anticoagulant or antiplatelet medication when taking a medical history. Eighty-seven percent (*n* = 137) agreed that they knew who to contact to get further information about patients’ medication, however only 50% (*n* = 79) agreed that it was straightforward to obtain the information. Seventy percent (*n* = 109) of participants agreed that they had sufficient time to ask patients about anticoagulant or antiplatelet use.

### Assessing bleeding risk

Ninety percent (*n* = 142) of respondents agreed that they knew how to assess the risk of bleeding complications associated with required dental procedures and 71% (*n* = 111) felt that they had sufficient time within routine appointments to assess a patient’s individual risk of bleeding. Ninety-eight percent (*n* = 154) of respondents felt that it was important to them to assess patient’s individual risk of bleeding prior to dental procedures.

Regarding the COM-B model component of opportunity, respondents working in the PDS more frequently felt they had sufficient time to assess bleeding risk than respondents working in the GDS (F = 8.52, *p* < 0.000) (Table 2).

**Table 2:** ANOVA beliefs and age, setting, years qualified.

Current practice	Beliefs	Mean difference	Standard error	F	df	*p*	Tukey post-hoc *p* value
Setting	Sufficient time to assess bleeding risk (14.4)	1.00	0.31	8.52	2, 152	<0.000	PDS vs GDS *p* = 0.004
	Feeling it was straightforward to liaise with medical colleagues to confirm INR readings (19.3.4)	1.35	0.34	9.23	2, 153	<0.000	PDS vs GDS *p* < 0.000
	Time to carry out packing and suturing (23.3)	1.43	0.36	11.77	2, 152	<0.000	PDS vs GDS *p* < 0.00
		2.40	0.76	11.77	2, 152	<0.000	PDS vs other *p* = 0.006
	Remuneration for packing and suturing (23.4)	1.19	0.31	7.49	2, 149	0.001	PDS vs GDS *p* < 0.001
	Time to liaise with colleagues in secondary care (25.3)	1.87	0.38	17.14	2, 153	<0.000	PDS vs GDS *p* = 0.000
		3.04	0.82	17.14	2, 153	<0.000	Other vs GDS *p* = 0.001

### Managing patients taking DOACs

Ninety percent (*n* = 141) of respondents felt that they understood how to manage patients taking DOAC medication and 91% (*n* = 143) felt that they had the skills to do so. Seventy-seven percent (*n* = 120) of respondents felt it was straightforward to obtain up-to-date information about DOACs. Seventy percent (*n* = 110) of respondents felt comfortable advising patients to miss their morning dose of apixaban or dabigatran when appropriate and 69% (*n* = 108) felt comfortable advising patients to delay their morning dose of rivaroxaban or edoxaban when appropriate.

### Managing Patients taking Warfarin or VKA

Ninety-two percent (*n* = 144) of respondents stated that they know when a patient’s INR should be checked before carrying out a procedure which is likely to cause bleeding for patients with a stable INR, and for those with an unstable INR (85%, *n* = 134). Sixty-two percent (*n* = 98) felt that they could rely on patients to report their most recent INR accurately. Fifty-one percent (*n* = 80) felt it was straightforward to liaise with medical colleagues to confirm a patient’s most recent INR before carrying out a procedure likely to cause bleeding. Checking INR readings prior to carrying out procedures likely to cause bleeding was routine practice for 94% (*n* = 148) of respondents.

Regarding the COM-B model component of opportunity, respondents working in the PDS more frequently felt it was straightforward to liaise with medical colleagues to confirm INR readings, than respondents working in the GDS (F = 9.23, *p* < 0.000).

### Managing patients taking a LMWH

Forty-one percent (*n* = 64) of respondents felt that they knew how to appropriately manage patients taking low doses of LMWH and felt confident to do so. Forty-two percent (*n* = 66) of respondents agreed that they could access timely advice from a patient’s prescribing clinician when deciding how to manage these patients.

### Carrying out haemostatic packing and suturing

Ninety-one percent (*n* = 144) of respondents understood how to effectively carry out haemostatic packing and suturing and 64% (*n* = 99) felt they had sufficient time to carry out packing and suturing during an appointment. Twelve percent (*n* = 17) felt there was sufficient remuneration from the SDR (Statement of Dental Remuneration) * to carry out packing and suturing in general practice.

Respondents working in the PDS more frequently felt they had sufficient time to carry out packing and suturing, than colleagues in the GDS, (F = 11.77, *p* < 0.000) and felt there was sufficient remuneration for packing and suturing from the SDR, (F = 7.49, *p* < 0.001).

Regarding the COM-B model component of opportunity, respondents who worked in the PDS more frequently felt they had the opportunity to carry out packing and suturing, than dental professionals working in the GDS (F = 11.77, *p* < 0.000) and other clinical settings (F = 11.77, *p* < 0.000).

### Referrals to secondary care for patients taking anticoagulant or antiplatelet drugs

Ninety percent (*n* = 141) of respondents agreed that they understood when it was appropriate to contact a colleague in secondary care to discuss the most appropriate management for the patient.   Forty-four percent (*n* = 70) agreed they had sufficient time in primary care settings to contact colleagues in secondary care to discuss management. Ninety-five percent (*n* = 147) agreed it was important to them only to refer patients to secondary care if there was a concern about safely managing patient care in primary care settings.

Regarding the COM-B component of opportunity, respondents working in PDS and other clinical settings more frequently felt they had sufficient time to contact secondary care colleagues (F = 17.14, *p* < 0.000), (F = 17.14, *p* < 0.000), than respondents working in the GDS.

There were no statistically significant differences noted between respondents’ age, sex, location, number of years qualified and beliefs.

*The Statement of Dental Remuneration (SDR), is a document produced by the Scottish Government, outlining NHS fees for provision of treatment in primary dental care settings.

### Linear regression

Linear regression was carried out to explore the relationship between the clinical behaviours and the predictors posited by the COM-B model. Behaviours included taking a medical history, assessing bleeding risk, managing patients taking DOACs, managing patients taking warfarin or another vitamin K antagonist, managing patients taking a LMWH, carrying out haemostatic packing and suturing and referrals to secondary care for patients taking anticoagulant or antiplatelet drugs (Table 3).

**Table 3:** Regression analysis.

Behaviour	Significant predictors	R^2^	F	df	p
Taking a medical history	• Respondent’s intention to ask about current anticoagulant or antiplatelet use and medical conditions	0.24	15.2	3	<0.00
	• Knowledge regarding who to contact for further information about antiplatelet or anticoagulant medication regimes				
Assessing bleeding risk	• Capability	0.57	93.0	2	<0.00
	• Importance they placed on assessing a patient’s individual risk of bleeding complications				
Managing patients taking DOACs	• Motivation	0.14	22.3	1	<0.00
Carrying out packing and suturing	• Gender	0.20	11.4	3	<0.00
	• Capability				
	• Number of years since qualification				
Referring to secondary care	• Capability	0.14	24.0	1	<0.00

Independent variables included: demographic information (sex, age, location, setting, years qualified) and beliefs (capability, opportunity, and motivation), related to each behaviour.

#### Thematic analysis

A total of 126 free-text responses were received: 28 responses related to taking a medical history, 10 responses related to assessing bleeding risk, 22 responses related to DOACs, 14 responses related to warfarin, 17 responses related to LMWH, 26 responses related to packing and suturing, and 9 responses related to referrals.

Analysis of free text responses highlighted a number of themes related to each of the behaviours:

## Taking a medical History

### GPs and other medical practitioners

Thematic analysis revealed that respondents experienced difficulty in corresponding with general medical practitioners (GMPs), to gain more information about the patient’s medical conditions and medications. Gaining timely feedback and advice from GMPs was difficult, and although respondents found secondary care consultants and specialists more accessible, the process is time consuming, and response time is often slow.

*‘It is extremely difficult if not impossible to get in touch with the patient’s GP, they never get back to me, except Consultants. - (GDP Associate)*

Respondents expressed concerns about the lack of knowledge of most medical practitioners regarding dental procedures and implications of anticoagulant or antiplatelet medication, suggesting that most medical colleagues do not feel comfortable providing advice due to their own lack of education and training on this topic.

*‘How to communicate altering medications with medical colleagues is a bit unclear - do we just tell them we’re doing this? ask their permission? Or ask their advice? I often get the response that they don’t know enough to advise or comment so think prescribers also need education’. - (Dental Consultant)*.

### Medical records

The majority of general dental practitioners reported that they did not have access to electronic medical records, clinical letters, referrals and medication lists, making it difficult to verify medication prior to dental procedures. Dental professionals in PDS and salaried dental services found it more straightforward to check anticoagulation medication for patients due to access to electronic care records.

*‘I think it would be sensible to allow GDPs the access to ECS (Emergency Care Summary) at least to check meds’. - (Specialty Dentist)*

### SDCEP Guidance

Clarity is often required from medical practitioners if patients’ drug regimens differ from recommendations provided by the SDCEP guidance. Dentists acknowledge clinical judgement is also required with use of the SDCEP guidance.

*‘I do always need to clarify guidelines with SDCEP. Sometimes, the patient’s drug regimen may differ from that suggested in the guidelines and then I struggle to know what to do’ - (Associate Dentist)*

## Assessing bleeding risk

### Time constraints

Due to time constraints with appointments in general practice, it can be difficult to assess bleeding risks associated with individual dental procedures and the individual risk to the patient.

*My role in PDS allows me to arrange long assessment appointments - it would be difficult for a GDP in high street practice to assess bleeding risk in a 5* *min exam appointment’. - (Specialist Practitioner)*

## Managing patients taking DOACs

### Following SDCEP guidance

Several respondents stated they do not always follow the SDCEP guidance for procedures deemed ‘low risk’, due to conflicting advice from specialists, who have recommended patients miss morning doses of anticoagulant medication prior to any extraction, regardless of the associated bleeding risk, and are aware a number of their colleagues also adopt this management strategy.

*‘I find that most of my colleagues routinely miss the AM dose of apixaban when doing extractions, whether simple and 1–3 teeth or complex, contrary to what SDCEP seems to guide us so, I tend to follow the consensus and also miss the AM dose for patients on DOACs needing an extraction whether low or high risk for bleeding’. - (Associate Dentist)*

Most respondents who left a comment noted that they lacked confidence to stop/alter medication doses without first speaking to colleagues in secondary care settings, GPs or consulting the SDCEP guidance.

*As it is fairly uncommon for me to be planning surgery/extractions on such patients I would not want to rely on my recollection of the guidance & would refer to SDCEP guidance to guide my assessment & management strategies. - (Associate Dentist)*

## Managing patients taking Warfarin or another vitamin K antagonist

### INR records

Although in the majority of cases dental professionals are able to confirm INR readings via patients’ INR recording books, sometimes they rely on the patient’s word that INR has been tested and is within correct values. Qualitative comments revealed that some respondents felt that medical colleagues sometimes underestimate the importance of timely INR checks prior to dental treatment, and this can result in a delay with treatment.

*‘Access to INR checks can be difficult and delay treatment’. - (Consultant Community Dental Services)*

## Managing patients taking a LMWH

### Lack of knowledge and confidence

Respondents acknowledged the rarity of encountering patients taking LMWHs and their lack of experience in managing these patients. Further guidance would be welcomed by dental professionals, to aid management.

*‘I have not treated patients taking heparin, so I am unaware of the standard advice. I would however consult SDCEP guidance if required to do so, or seek advice from a special care dentist if it did not provide the information required’. - (Associate Dentist)*

## Carrying out haemostatic packing and suturing

### Barriers to packing and suturing

A number of dental professionals raised concerns regarding the lack of remuneration available from the NHS in general practice for packing and suturing. They highlighted the longer appointments required to provide this service and the knowledge and skills required to complete packing and suturing. A number of respondents also stated they would not carry out packing and suturing for all patients and base this decision on the individual risk assessment of the planned procedure and patient.

*‘If I have identified a patient as having a bleeding risk, I make sure I book a longer appt to allow packing, suturing and haemostasis. The SDR does not remunerate adequately for this’. - (Associate Dentist)*

## Referrals to secondary care for patients taking anticoagulant or antiplatelet drugs

### Referrals and advice

Dental professionals expressed difficulty in reaching dental practitioners and specialists in community dental services or hospital settings for advice and guidance.

*‘Getting hold of colleagues in the hospitals quickly to discuss is almost impossible’. (Associate Dentist)*

Dental professionals in rural settings also expressed apprehension about carrying out complex procedures due to the distance away from secondary care settings, in case of complications.

*‘I would not feel comfortable doing a more complex procedure on a patient on anticoagulants. Due to the distance from my practice to a dental hospital. I would usually refer them’. - (Associate Dentist)*

## Discussion

The SDCEP guidance on managing dental patients taking anticoagulants or antiplatelet drugs was updated in 2022, taking into consideration the increased prevalence of DOAC prescribing and uncertainty about managing treatment for patients taking LMWHs. The updated guidance includes recommendations and advice for the newest DOAC, edoxaban, and the LMWHs, and updated information to support bleeding risk assessment.

In line with the TRiaDS framework [19] for supporting the development and implementation of SDCEP guidance, the purpose of this project was to investigate the current practice and beliefs of dental professionals surrounding the management of dental patients taking anticoagulant and antiplatelet drugs. The results provide an overview of these before the updated guidance was published and identify potential barriers to following the guidance recommendations in primary care settings.

The results of the pre-publication survey highlighted that there were discrepancies between current practice and practice recommended in the 2015 edition of the guidance. The results highlighted very good awareness (100% of respondents stated they were aware of the guidance), and good adherence (77% of respondents stated they always base their practice on the guidance). However, the results also highlighted potential areas that could be targeted for improvement.

Whilst the SDCEP guidance advises that dental professionals should ask patients about their current or planned use of anticoagulants or antiplatelet drugs and other medications, when taking or confirming their medical history [1], only 59% of respondents (*n* = 93) always asked specifically about the use of anticoagulant or antiplatelet medications. This suggests that further support related to taking a medical history is required.

The SDCEP guidance also advises that for dental procedures with a low associated bleeding risk, anticoagulant medication regimes should not be altered, due to the increased risk of thromboembolic events [1]. However, although 90% (*n* = 141) of respondents felt that they understood how to manage patients taking DOAC medication, only 38% always follow SDCEP guidance advice not to interrupt DOAC medication regimes for procedures with a low associated risk of bleeding. The free text responses offer further insight, revealing that a number of respondents do not always follow the SDCEP guidance for procedures deemed ‘low risk’ due to conflicting advice from local specialists, who have recommended patients miss morning doses of DOAC medication prior to any extractions, regardless of the associated bleeding risk. Respondents also stated they were aware that several colleagues also adopt this management strategy.

The evidence supporting altering DOAC medications and altering medication regimes based on bleeding risk in the guidance document is limited, due to the low quality of some studies and the paucity of evidence relating to dental procedures with a higher risk of bleeding. The 2015 edition of the guidance acknowledged the lack of supporting evidence from dental studies. This may contribute to lack of compliance with guidance recommendations surrounding DOACs and dental procedures as noted in the qualitative data. Existing research and meta-analyses have also concluded that evidence regarding DOAC management in a dental setting is limited and studies are of low quality [20, 21].

Existing research also found that adherence to SDCEP guidance was low regarding DOAC management, with compliance ranging from 25–57% in one study. The study concluded that the complexity of guidance recommendations may contribute to non-compliance. The study findings also supported the guidance recommendation for audit and regular staff education to improve compliance and staff knowledge [22].

Respondents highlighted a lack of confidence, knowledge, experience, and skills in managing patients taking LMWH. This was evident from both quantitative and qualitative analysis. Free text responses acknowledged the rarity of encountering patients on LMWH and the need to contact prescribing clinicians for advice. Respondents highlighted that they would welcome further guidance surrounding the dental management of patients taking LMWH. Only 41% (*n* = 64) of respondents felt that they knew how to appropriately manage patients taking low doses of LMWH and felt confident to do so. The second edition of the SDCEP guidance [1] has been updated to include management advice for patients on low prophylactic doses and higher doses of LMWH, to support dental professionals.

Statistical analysis revealed that the only demographic variable where there were significant differences in beliefs was setting (GDS, PDS, other). Regarding the COMB domain of opportunity, dental professionals working in PDS had greater opportunity to assess bleeding risk, carry out packing and suturing and liaise with medical professionals, than colleagues in GDS. Time pressures, workload, and the environment of general medical practice [23] can often result in barriers to implementation of evidence-based practice, this is applicable in both general medical and dental settings. Greater opportunities and support for dental professionals in GDS are required to ensure guidance can be implemented into general dental practice. Regarding the COM-B component of opportunity, interventions which provide greater financial support and time made available in general dental practice for dental professionals, would aid guidance implementation and behaviour change [14].

Thematic analysis of free text responses identified time, remuneration and access to electronic health records and patient medication lists as barriers to guidance implementation and adherence to SDCEP guidance.

These barriers to guidance implementation noted from free text responses and discrepancies noted in current practice support the notion that guidance publication alone is unlikely to result in desired changes in practice by healthcare professionals [24]. Whilst barriers such as insufficient time and remuneration cannot be addressed by SDCEP, educational support could help address barriers to implementation by improving knowledge and further developing skills surrounding the management of patients on anticoagulant or antiplatelet drugs. Studies from the existing evidence base have also highlighted the need for further education of both medical and dental practitioners in using evidence-based guidelines surrounding dental treatment for patients on anticoagulant or antiplatelet therapy [25]. Educational programmes and workshops related to anticoagulants and antiplatelets have been shown to increase awareness of practitioners to update their knowledge and practice regarding management of dental patients taking anticoagulant or antiplatelet drugs [26]. In order to address barriers such as time and remuneration, wider stakeholder and governmental involvement would be required, to provide further financial support to dental professionals working in general dental services.

The findings of this project contribute to the current body of evidence surrounding dental professionals’ beliefs and current practice surrounding the dental management of patients on anticoagulant and antiplatelet drugs, highlighting potential barriers to guidance implementation in primary care. However, several limitations are recognised. Whilst the use of questionnaires offers an objective means of collecting information about respondents’ knowledge, beliefs and behaviour [27], there are often limitations with their use in research. The potential for response bias must be considered, as the dental professionals who submitted responses may be more engaged with the SDCEP guidance and make more regular use of it in their daily practice. Recall bias is a further limitation that must be considered, in terms of the subjective nature of respondents’ self-reported knowledge, motivation, opportunity and capability. The findings of the questionnaire may not be generalisable to the general population in the UK as respondents were recruited through the NES Portal and VDP training hub, and practice may differ between countries.

Future work will involve a post-publication questionnaire, to be disseminated to all dental professionals following publication of the second edition of the guidance. Further education and support could be provided, to support implementation of the guidance into primary care settings and to provide support, if the post-publication questionnaire provides no evidence of a change in beliefs and current practice surrounding LMWH use and assessing bleeding risk.

## Conclusion

In order to support the implementation of the updated SDCEP guidance, the aim of the project was to determine barriers to compliance and to identify training needs of dental professionals.

Analysis of the pre-publication questionnaire results concluded that there is very good awareness of the guidance in primary care settings, with good adherence to the advice and guidance suggested. However, further educational support may be required regarding the management of patients taking low molecular weight heparins and the management of patients on DOAC medication.

## References

[1] [Internet], SDCEP Clinical Effectiveness Programme, Management of dental patients taking anticoagulants or antiplatelet drugs, 2022. Available from: https://www.sdcep.org.uk/published-guidance/anticoagulants-andantiplatelets/. Accessed 18 July 2022.

[2] [Internet] Randall C, Surgical Management of the Primary Care Dental Patient on Antiplatelet Medication: North West Medicines Information Centre, 2010. Available from www.app.dundee.ac.uk/tuith/Static/info/antiplatelet.pdf. Accessed 18 July 2022.

[3] Van EsN, Coppens M, Schulman S, Middeldorp S, Büller H (2014). Direct oral anticoagulants compared with vitamin K antagonists for acute venous thromboembolism: evidence from phase 3 trials. Blood.

[4] Ruff C, Giugliano R, Braunwald E, Hoffman E, Deenadayalu N, Ezekowitz M (2014). Comparison of the efficacy and safety of new oral anticoagulants with warfarin in patients with atrial fibrillation: a meta-analysis of randomised trials. Lancet.

[5] [Internet], National Institute for Health and Care Excellence (NICE), Assumptions used in estimating a population benchmark. 2012. Available at: https://www.nice.org.uk/usingguidance/commissioningguides/anticoagulationtherapyservice/popbench.jsp. Accessed 18 July 2022.

[6] Afzal S, Zaidi S, Merchant H, Babar Z, Hasan S (2021). Prescribing trends of oral anticoagulants in England over the last decade: a focus on new and old drugs and adverse events reporting. J Thrombosis Thrombolysis.

[7] Vinogradova Y, Coupland C, Hill T, Hippisley-Cox J (2018). Risks and benefits of direct oral anticoagulants versus warfarin in a real world setting: cohort study in primary care. BMJ.

[8] [Internet], British National Formulary, Chapter 2 cardiovascular system, 2.9 Antiplatelet drugs, available at: https://openprescribing.net/bnf/0209/. Accessed 18 July 2022.

[9] Thachil J (2016). Antiplatelet therapy – a summary for the general physicians. Clin Med.

[10] [Internet] Paraschiv C, Esanu I, Ghiuru R, Manea P, Munteanu D, Gavrilescuet C. Dental implications of the new oral anticoagulants. 2015. Available from: http://www.rjor.ro/wp-content/uploads/2015/12/DENTAL_IMPLICATIONS_OF_THE_NEW_ORAL_ ANTICOAGULANTS.pdf. Accessed 18 July 2022.

[11] Green L, Tan J, Morris J, Alikhan R, Curry N, Everington T (2018). A three-year prospective study of the presentation and clinical outcomes of major bleeding episodes associated with oral anticoagulant use in the UK (ORANGE study). Haematologica.

[12] [Internet] Randall C. Surgical Management of the Primary Care Dental. Patient on Warfarin: North West Medicines Information Centre. 2007; Available from: www.app.dundee.ac.uk/tuith/Static/info/warfarin.pdf. Accessed 18 July 2022.

[13] Kelly N, Nic Íomhair A, Hill S, McKenna G, O’Carolan D, Cleary G (2021). Perceptions of General Dental Practitioners in Northern Ireland on the clinical management of patients taking Direct Oral Anticoagulants (DOACs). J Ir Dent Assoc.

[14] Michie S, Atkins L, West R. The behaviour change wheel. [S.l.]: Silverback Publishing; 2014.

[15] Ursachi G, Horodnic I, Zait A (2015). How reliable are measurement scales? External factors with indirect influence on reliability estimators. Procedia Econ Financ.

[16] Jafari M, Ansari-Pour N (2019). Why, when and how to adjust your *p* values?. Cell J..

[17] Kiger M, Varpio L (2020). Thematic analysis of qualitative data: AMEE Guide No. 131. Med Teach.

[18] Braun V, Clarke V (2006). Using thematic analysis in psychology. Qualitative Res Psychol.

[19] Clarkson J, Ramsay C, Eccles M, Eldridge S, Grimshaw J, Johnston M (2010). The translation research in a dental setting (TRiaDS) programme protocol. Implement Sci.

[20] Manfredi M, Dave B, Percudani D, Christoforou J, Karasneh J, Diz Dios P (2019). World workshop on oral medicine VII: direct anticoagulant agents management for invasive oral procedures: a systematic review and metaanalysis. Oral Dis.

[21] Fortier K, Shroff D, Reebye U (2018). Review: an overview and analysis of novel oral anticoagulants and their dental implications. Gerodontology.

[22] Woolcombe S, Ball R, Patel J (2022). Managing direct oral anticoagulants in accordance with the Scottish Dental Clinical Effectiveness Programme guidance for patients undergoing dentoalveolar surgery. Br Dent J.

[23] Zwolsman S, te Pas E, Hooft L, Waard M, van Dijk N (2012). Barriers to GPs’ use of evidence-based medicine: a systematic review. Br J Gen Pract.

[24] Elouafkaoui P, Bonetti D, Clarkson J, Stirling D, Young L, Cassie H (2015). Is further intervention required to translate caries prevention and management recommendations into practice?. Br Dent J.

[25] Shah A, Khalil H, Alshahrani F, Khan S, AlQthani N, Bukhari I (2015). Knowledge of medical and dental practitioners towards dental management of patients on anticoagulant and/or anti-platelet therapy. Saudi J Dent Res.

[26] Linnebur S, Ellis S, Astroth J (2007). Educational practices regarding anticoagulation and dental procedures in U.S. Dental Schools. J Dent Educ.

[27] Sapsford R. Survey research. London: SAGE Publications; 1999.

